# Phase Field Investigation on Grain Boundary Migration Affected by Intergranular Mobile Pores in UO_2_ Fuels

**DOI:** 10.3390/ma19153174

**Published:** 2026-07-24

**Authors:** Caiyan Liu, Hongliang Du, Zhuang Miao, Jiahui Qu, Jiaxuan Si, Tao Peng, Lu Wu, Jing Zhang

**Affiliations:** 1 Multifunctional Electronic Ceramics Laboratory, College of Engineering, Xi’an International University, Xi’an 710077, China; liucaiyan2019@yeah.net (C.L.); duhongliang@126.com (H.D.); miaozhuang@xaiu.edu.cn (Z.M.); 2 State Key Laboratory of Solidification Processing, Northwestern Polytechnical University, Xi’an 710072, China; qujiahui@mail.nwpu.edu.cn; 3 Nuclear Power Institute of China, Chengdu 610005, China; sjx@npic.ac.cn (J.S.); pengtaoustu@163.com (T.P.)

**Keywords:** pore, grain boundary, grain growth, microstructure of UO_2_ fuel, phase field simulation

## Abstract

The steep radial temperature gradients developed in UO_2_ fuels during reactor operation can drive pore migration, making pore–grain boundary (GB) coupled migration an important mechanism governing microstructural evolution. Although pores are generally regarded as pinning features that hinder GB migration, the conditions under which mobile pores retard, co-migrate with, or promote GB migration remain poorly understood. In this study, we develop a phase field model coupling vapor-transport-driven pore migration and curvature-driven grain growth to investigate the coupled migration behavior between intergranular mobile pores and GBs. The simulations first focus on an idealized source-term-free system to isolate the effect of pore–GB migration coupling from irradiation-induced pore generation and growth. The results show that the effect of pores on GB migration depends on the relative migration rate of pores and GBs, which is determined by both the pore-to-GB mobility ratio and the corresponding driving-force ratio. When pores migrate more slowly than GBs, they retard GB migration and exhibit an effective pinning effect. In contrast, sufficiently mobile pores can co-migrate with GBs and promote apparent GB migration when the pore migration rate exceeds that of the GBs. Furthermore, to illustrate the regulating effect of pores on GB migration in UO_2_ under a temperature gradient, we perform additional simulations under continuous irradiation, in which pores nucleate spontaneously, migrate along the temperature gradient, and interact with GBs, in agreement with experimental observations. Based on the simulated GB migration behavior, we construct a regime map that distinguishes pinning/retardation, weak interaction, and pore-assisted migration regimes. This work provides a mechanistic phase field interpretation of pore–GB coupled migration and offers insight into microstructural evolution in porous oxide fuel materials.

## 1. Introduction

Many physical and mechanical properties of materials depend on the grain morphologies, including grain size, pattern, and alignment [1]. The evolution of grain morphology through grain boundary (GB) migration is influenced by solute segregation, obstacle particles, as well as thermal and stress conditions [2,3]. In cases where mobile pores act as obstacle particles, such as in porous materials or irradiated UO_2_ fuels, the interaction between the mobile pores and the GBs can lead to complex changes in the microstructure of grains [4,5]. Understanding the pore–GBs interaction is essential for the development and optimization of nuclear fuels with desired properties.

Uranium dioxide (UO_2_) serves as the main fuel in commercial light water reactors. During reactor operation, the fuel is exposed simultaneously to intense radiation damage and steep thermal loading. Its low thermal conductivity allows a large temperature gradient to develop across the pellet. This gradient drives microstructural restructuring and sets up thermal stresses that promote cracking and the directional migration of pores and other defects [6]. Consequently, the evolution of the UO_2_ microstructure becomes a key factor governing fuel performance and integrity. Highly irradiated porous UO_2_ fuel pellets commonly exhibit pronounced radially heterogeneous microstructures, including a central cavity, an adjacent columnar grain region oriented towards the fuel center, followed by an equiaxed grain structure region and an unstructured region at the periphery [7]. This characteristic radial partitioning is generally attributed to the severe radial temperature gradients established during operation, which provide strong thermally driven forces for microstructural evolution. Under such conditions, thermally activated GBs and mobile pores interact: pores segregate to GBs to form pore–GB complexes that can migrate collectively under the temperature gradient and associated thermomechanical stresses [6,8]. Although pore–GB interactions have been implicated in promoting columnar grain formation [9], there remains insufficient understanding of how the collective movement of pore–GB complexes affects GB migration and the formation of distinctive microstructures.

Theoretical models for GB migration in the presence of obstacles are formulated based on the treatment of obstacles as either mobile or immobile [10,11]. When pores are treated as immobile obstacles, the classical Zener model is utilized to describe the pinning effect on GB migration. The pinning force exerted on GBs by pores is associated with pore shape, size, contact angle, and density [11,12]. If pores attached to GBs are considered mobile, either a pore-controlled GB migration model or a boundary-controlled GB migration model is adopted, depending on the relative mobility of pores versus GB. The pore-controlled GB migration model governs when the mobility of pores is lower compared to that of a GB and thus slows down the migration speed of a GB; in this case, the pores are treated as rigid bodies capable of moving along with GBs. Conversely, the boundary-controlled GB migration model is applicable when pore mobility greatly exceeds that of the GB; in this case, pores detach from GBs and have a negligible effect on GB migration [13,14]. As porosity increases, the GB migration mechanism gradually shifts from being controlled by boundaries to being controlled by pores [15,16,17].

The aforementioned theoretical models offer valuable insights into the GB migration behavior involving mobile pores under the rigid-body assumption [14]. However, these models do not accurately depict the reality of pore morphologies as the shapes and characteristics of pores in actual materials can be highly complex and variable, and the existing theoretical models tend to simplify these details. Some sharp-interface models have also been utilized to study the collective movement of pores and GBs in porous polycrystalline solids [18,19], but they encounter challenges in tracing the pore–grain boundary interface. In contrast, the phase field method employs a diffusive interface to address issues related to GB migration involving pore movement and is capable of capturing subtle morphological changes without explicit tracing of interfaces [16,17,20]. K. Ahmed et al. [15,16] utilized phase field methods to reveal the pinning effect of pores on GBs through surface diffusion under isothermal conditions in porous UO_2_. Biner et al. [21] also employed phase field methods to investigate the collective movement of pore–GB complexes and the spatial partitioning of pores affected by GBs. Their work assumed that pores act only as a drag on GBs and examined coupled pore–GB migration only in two limiting regimes of GB mobility, while neglecting the effect of directional pore migration on GB motion. Overall, the studies of GB migration behavior involving pores are complex and ongoing, and they have not yet accounted for the development of columnar grains observed in UO_2_ fuel under steep temperature gradients.

Pore–GB interactions are also influenced by the pore migration mechanism, since different transport pathways can yield different pore mobilities and morphological evolution. In UO_2_ fuel, pores may migrate via vapor transport (evaporation–condensation), surface diffusion, or lattice diffusion, with the dominant mechanism determined by pore size and the thermal-stress conditions. Surface diffusion dominates at the nanoscale pores, vapor transport dominates at the micron scale, and lattice diffusion prevails at extremely high temperatures. The vapor transport mechanism is characterized by significant pore mobility resulting from steep temperature gradients; it is the dominant mechanism for pore migration in regions where grains undergo columnar grain formation [22,23,24]. The migration of pores via different mechanisms inevitably affects the GB migration in porous crystalline materials.

In this study, by integrating the pore migration via vapor transport mechanism with curvature-driven grain growth, we develop a phase field model to investigate grain growth involving mobile pores driven by a temperature gradient. The phase field model formulation and numerical implementation are introduced in Section 2. In Section 3, we apply various pore-to-GB mobility ratios to a bi-crystal system containing a pore–GB complex located in a high-temperature field with a steep temperature gradient. The grain growth kinetics via collective migration of the pore–GB complex are then investigated. Finally, conclusions are summarized in Section 4.

## 2. Phase Field Models

### 2.1. Phase Field Model of Pore–GB Interaction

To reproduce the interaction behavior between pores and grain boundaries in porous uranium dioxide, we developed a multiphase field framework that couples curvature-driven grain growth with pore migration governed primarily by vapor transport. Within this framework, each grain is described by one member of a family of non-conserved order parameters $\eta_{\alpha }(r,t)$ (*α* = 1, 2……*N*, with *N* denoting the number of grains). Under the assumption of isotropic GB properties, a given order parameter equals 1 inside its own grain and 0 elsewhere—including in neighboring grains and in pores. The pores are described by a conserved fractional solid density, *ρ*(*r*,*t*), which equals 0 within the pore and 1 inside the matrix phase. In the matrix–pore interface region, this density changes smoothly from 1 at the matrix to 0 in the pore. All order parameters and field variables change smoothly, rather than abruptly, across their respective interfaces. Microstructural evolution is driven by the tendency of these variables to reduce the system total free energy, which is expressed as a functional of the field variables:

(1) $$F=\int_{V}\left(f_{gb}(\eta ,\nabla \eta )+f_{p}(\rho ,\nabla \rho ,\eta )\right)dV$$

The polycrystalline free-energy density $f_{gb}$ is written as

(2) $$f_{gb}(\eta ,\nabla \eta )=\frac{\kappa_{\alpha \beta }}{2}\sum_{\alpha =1}^{N}\sum_{\beta =\alpha +1}^{N}\left|\nabla \eta_{\alpha }\cdot \nabla \eta_{\beta }\right|+H_{\alpha \beta }\sum_{\alpha =1}^{N}\sum_{\beta =\alpha +1}^{N}\eta_{\alpha }\eta_{\beta }$$

Here the first (gradient) term on the right-hand side supplies the interfacial free energy, with $\kappa_{\alpha \beta }$ (units J/m) as its gradient coefficient, while the second term is a double-obstacle potential barrier function whose barrier height is set by $H_{\alpha \beta }$ (units J/m^3^). These two coefficients are linked to the grain boundary energy $\gamma_{gb}$ and grain boundary width *W* through $\kappa_{\alpha \beta }=\gamma_{gb}W$ and $H_{\alpha \beta }=\gamma_{gb}/W$.

The pore free energy density $f_{p}$ is given by

(3) $$f_{p}(\rho ,\nabla \rho ,\eta )=B\rho^{2}{\left(1-\rho \right)}^{2}+\frac{\kappa_{\rho }}{2}{\left|\nabla \rho \right|}^{2}+Cf^{\mathrm{int}}(\rho ,\eta )$$

In this expression, the first term is a double-well chemical potential, the second (gradient) term represents the surface energy, with $\kappa_{\rho }$ as its gradient coefficient (J/m), and the third term describes the interaction energy between grain boundaries and pores. *B* and *C* are material constants.

The interaction term is written as a summation of the interaction functions $f_{\alpha }^{\mathrm{int}}$ over all grains:

(4) $$f^{\mathrm{int}}(\rho ,\eta )=\sum_{\alpha =1}^{N}f_{\alpha }^{\mathrm{int}}(\rho ,\eta_{\alpha })$$

The interaction operates only at a pore–grain interface (0<ρ<1) and vanishes inside a pore (ρ=0) or grain (ρ=1, $\eta_{\alpha }=1$). The interaction function can be written in the form proposed in [25]:

(5) $$f_{\alpha }^{\mathrm{int}}(\rho ,\eta_{\alpha })=\rho^{2}(1-\rho )\eta_{\alpha }(1-\eta_{\alpha })+\frac{1}{3}(\rho^{3}-1)\eta_{\alpha }(1-\eta_{\alpha })$$

The evolution of the conserved order parameter *ρ* follows the time-dependent Cahn–Hilliard equation:

(6) $$\frac{\partial \rho }{\partial t}=\nabla \cdot \left(M_{p}\nabla \frac{\delta F}{\delta \rho }\right)-P_{v}(r,t)$$

where $M_{p}$ is the pore mobility, $M_{p}=D_{v}/k_{B}T$, $D_{v}$ is the diffusion coefficient of vacancy, $D_{v}=D_{v0}\exp(-E_{v}^{m}/k_{B}T)$, $E_{v}^{m}$ is the migration energy and $D_{v0}$ is the prefactor. Upon activation of a cascade event, vacancies are introduced through the source term *P_v_*(*r*,*t*), which represents irradiation-induced defect generation and enables spontaneous pore nucleation. The source term *P_v_*(*r*,*t*) is formulated as

(7) $$P_{v}(r,t)=\left\{\begin{array}{l} R_{2}V_{G}\;(\rho \geq 0.8\;\mathrm{or}\;R_{1}>\;P\mathrm{casc})\\ 0\;\;(\rho <0.8\;\mathrm{and}\;R_{1}\leq P\mathrm{casc}) \end{array}\right\}$$

Here *R*_1_ and *R*_2_ are random numbers uniformly distributed on [0, 1]. *P*_casc_ and *V_G_* denote the probabilities of cascade occurrence and vacancy generation, respectively. Defect production is restricted to the matrix phase (*ρ* ≥ 0.8), thereby preventing vacancy generation inside pre-existing pores. The source term *P_v_*(*r*,*t*) is set to zero in the bi-crystal and three-grain simulations (Section 3, Section 4.1 and Section 4.2) to isolate the intrinsic pore–GB interaction; it is activated only in Section 4.3, where continuous irradiation drives spontaneous pore nucleation under a temperature gradient.

The non-conserved field parameters evolve according to the time-dependent Allen–Cahn equation:

(8) $$\frac{\partial \eta_{\alpha }}{\partial t}=-\frac{2L}{n}\sum_{\beta =1}^{N}\left(\frac{\delta F}{\delta \eta_{\alpha }}-\frac{\delta F}{\delta \eta_{\beta }}\right)\;\;\alpha =1,\ldots N$$

where *n* is the local number of non-conserved phase fields $\eta_{\alpha }$, $n=\sum_{\alpha =1}^{N}\lambda_{\alpha }$, with $\lambda_{\alpha }=1$ when $0<\eta_{\alpha }<1$ and 0 otherwise. *L* is the kinetic coefficient governing grain boundary migration, satisfying $L\kappa_{\alpha \beta }=M_{b}\gamma_{gb}$, with $M_{b}$ the GB mobility.

Depending on the temperature distribution and pore size, pore migration in UO_2_ is generally governed by a combination of vapor transport (evaporation/condensation), surface diffusion, and bulk diffusion. In the columnar-grain region, the vapor-transport mechanism dominates, and the pore migration rate is determined as follows [8,26],

(9) $$v_{p}=a_{1}(a_{2}+a_{3}T+a_{4}T^{2}+a_{5}T^{3})T^{-\frac{5}{2}}\Delta H_{vap}p_{0}\exp\left(-\frac{\Delta H_{vap}}{RT}\right){\left(\frac{dT}{dr}\right)}_{p}$$

where $a_{1}$, $a_{2}$, $a_{3}$, $a_{4}$, and $a_{5}$ are constants whose values are listed in Table 1 [27]. $p_{0}$ is a pressure prefactor and $\Delta H_{vap}$ is enthalpy of evaporation of solid (J/mol), both derivable from vapor pressure data [8]. *R* is the ideal gas constant, ${\left(\frac{dT}{dr}\right)}_{p}$ is the temperature gradient inside the pore, and *r* refers to position. This temperature gradient-driven pore migration via the vapor transport mechanism (Equation (9)) is incorporated directly into Equation (6),

(10) $$\begin{array}{l} \frac{\partial \rho }{\partial t}=\nabla \cdot \left[M_{p}\nabla \left(2B\rho (1-\rho )(1-2\rho )+\kappa_{\rho }\nabla^{2}\rho )+C\sum_{\alpha =1}^{N}\frac{\partial f_{\alpha }^{\mathrm{int}}}{\partial \rho }\right)\right]\\ +\nabla \cdot (v_{p}\rho )-P_{v}(r,t) \end{array}$$

where the first term is the standard Cahn–Hilliard flux, $M_{p}\nabla (\frac{\delta F}{\delta \rho })$, where the chemical potential $\frac{\delta F}{\delta \rho }$ comprises three contributions: the bulk double-well term 2Bρ(1−ρ)(1−2ρ), the gradient-energy term $\kappa_{\rho }\nabla^{2}\rho$, and pore–GB interfacial-coupling term $C\sum_{\alpha =1}^{N}\frac{\partial f_{\alpha }^{\mathrm{int}}}{\partial \rho }$. The second term, $\nabla \cdot (v_{p}\rho )$, is the advective flux arising from vapor-transport-driven pore migration. *v_p_* is evaluated from the local temperature and thus retains the full exponential temperature dependence of vapor transport. Because *v_p_* scales with *dT*/*dr*, the advective term vanishes when the gradient is removed. Here *v_p_* is defined as positive along the direction of increasing temperature, consistent with the directional derivative (*dT*/*dr*)*_p_* in Equation (9). Because both the diffusive flux $M_{p}\nabla (\frac{\delta F}{\delta \rho })$ and the advective flux $\nabla \cdot (v_{p}\rho )$ appear in pure divergence (flux) form, they redistribute *ρ* only locally and cannot change the domain-integrated solid volume under the periodic boundary conditions used in all simulations. As a result, total solid volume is conserved exactly in the source-free simulations.

The pore surface energy $\gamma_{s}$, the pore interface width $W_{\rho }$, the gradient coefficient $\kappa_{\rho }$, and *B* are interrelated through the following relations.

(11) $$\gamma_{s}=\sqrt{\kappa_{\rho }B},\;\;\kappa_{\rho }=\gamma_{s}W_{\rho },\;\;B=\gamma_{s}/W_{\rho },\;\;W_{\rho }=\sqrt{\kappa_{\rho }/B}$$

### 2.2. Numerical Implementation

The simulations are performed on a two-dimensional computational grid. Although grain growth and its interaction with pores are inherently three-dimensional, our 2D model is oriented along the temperature-gradient direction and retains the GB curvature driving force through the in-plane curvature, thereby capturing the essential physics of the pore–GB interaction. A uniform rectilinear grid with 256 × 256 nodes is used for discretization, with ∆*x* = ∆*y* = *l*_0_ (*l*_0_ = 0.1 μm) for each grid. The time increment for each iteration step is *t*_0_ = 1.0 × 10^−4^ s. The finite difference method with an explicit forward Euler scheme is used in solving Equations (8) and (10). The following non-dimensional parameters are adopted: $t^{*}=t/t_{0}$, $l^{*}=l/l_{0}$, $\nabla^{*}=l_{0}\nabla$, ${M_{p}}^{*}=M_{p}Bt_{0}/{l_{0}}^{2}$, $B^{*}=B/B$, ${\kappa_{\rho }}^{*}=\kappa_{\rho }/B$, $C^{*}=C/B$, $L^{*}=LBt_{0}{l_{0}}^{2}$, ${\kappa_{\alpha \beta }}^{*}=\kappa_{\alpha \beta }/B{l_{0}}^{2}$, ${H_{\alpha \beta }}^{*}=H_{\alpha \beta }/B{l_{0}}^{2}$. *C* is the interaction energy parameter, and the reference energy density is *B* = 1.197 × 10^9^ J/m^3^ [15]. Parameters used in the phase field simulations are presented in Table 1.

The optimized algorithm is adopted and modified to enhance computational efficiency [30]. The periodic boundary conditions are applied. The mean interfacial energy of GBs and the mobilities of pores and GBs are taken into account. To simplify the calculations, average values of UO_2_ in the temperature range 1660–1940 °C are adopted.

## 3. Simulation Results

### 3.1. Model Reliability Analysis

**Figure 1:**
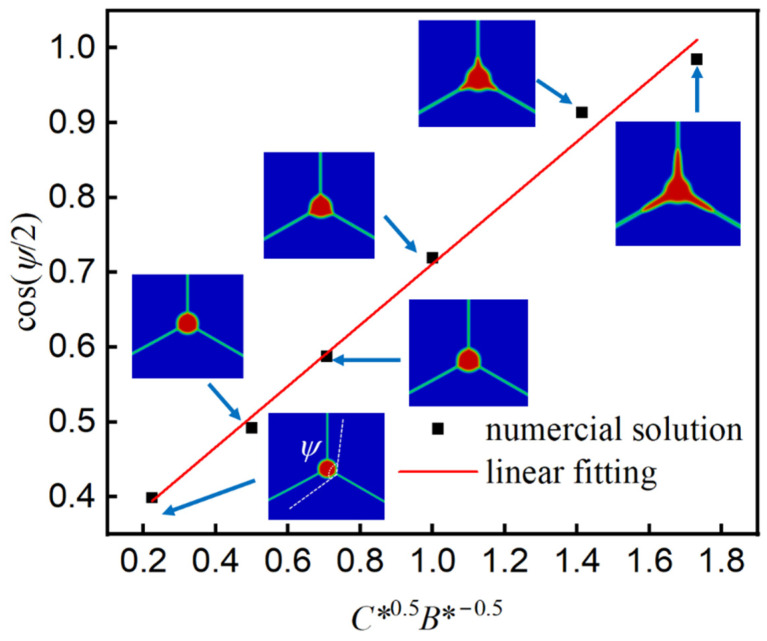
Comparison of the simulation results and analytical results regarding the morphological change in the dihedral angle under different model parameters.

To validate the accuracy of our model in describing the interactions between pores and GBs in our simulation, we simulate a triple junction attached to a single pore. The initially preset spherical pore adjusts during the phase field dynamic simulation and reaches a static equilibrium state under different values of *B*^∗^ and *C*^∗^, as depicted in Figure 1. The surface tensions at the boundaries are in static equilibrium [31] and satisfy Young’s equation:

(12) $$\cos(\frac{\psi }{2})=\frac{\gamma_{gb}}{2\gamma_{s}}$$

where $\gamma_{gb}$ and $\gamma_{s}$ are the grain boundary energy and surface energy per unit area, respectively, and *Ψ* is the dihedral angle, as shown in Figure 1.

When pores interact with GBs, the force exerted on the pore surface by the GB depends on both the interaction coefficient *C*^∗^ and the width of the grain boundary *W*:

(13) $$\gamma_{gb}\propto \sqrt{C^{*}W}$$

Substituting the relationships in Equation (11) into Equation (12), we obtain:

(14) $$\cos(\frac{\psi }{2})\propto \frac{\sqrt{C^{*}W}}{2\sqrt{\kappa_{\rho }B^{*}}}$$

It can be seen that cos(*Ψ*/2) has a linear relationship with $\sqrt{C^{*}/B^{*}}$ for given parameters of *W* and $\kappa_{\rho }$. That is, *Ψ*, which reflects the morphology of pores, will change accordingly.

The pores in triple junctions adjust themselves in response to the changing $\sqrt{C^{*}/B^{*}}$, as demonstrated in Figure 1. Specifically, the dihedral angle *Ψ* is measured directly from the phase field results. It is notable that cos(*Ψ*/2) shows a linear relationship with $\sqrt{C^{*}/B^{*}}$, which is consistent with the analysis of Young’s equation (Equation (12)). The physical models constructed in this study accurately capture the subtle morphological changes in pores that are influenced by GBs as described by Young’s equation. This ensures the reliability of these models in capturing the complex behaviors of pore–GB complexes by considering their morphological effects rather than treating them as rigid bodies.

Since the central claim of this work concerns dynamic pore-assisted GB migration, we further validate the two dynamic mechanisms in the coupled model independently. For pore-free curvature-driven grain shrinkage, the simulated grain area is shown to decrease linearly with time, consistent with the analytical curvature-driven solution (see Section 3.2 for details).

For isolated pore migration, the velocity predicted by Equation (9) has been validated against phase field simulations of isolated pores of varying aspect ratio under an imposed temperature gradient in our previous work [26], using the identical vapor-transport expression. The simulated migration velocity agreed closely with Equation (9) across the entire range tested. Together with the equilibrium morphology validation above, these results confirm that both dynamic mechanisms are validated independently.

### 3.2. Effect of Relative Mobility of Pores to GB on Grain Growth

The phase field simulation of grain growth, with pores attached to GB, is conducted in a two-dimensional (2D) bi-crystal system under periodic boundary conditions (PBCs). A centrosymmetric steep temperature gradient field and high temperature are imposed at the center of the simulation domain, driving the collective migration of pores and GBs.

At the beginning of the simulation, a centrosymmetric temperature distribution of $T=T_{1}+[(T_{2}-T_{1})/2]\left[\cos(D/D_{0})\pi +1\right]$ is positioned at the center of the simulation region, with the temperature decreasing from 2000 K (*T*_2_) at the center to 1900 K (*T*_1_) at the periphery, to replicate the temperature profile under operating conditions. Here, *D*_0_ = 240Δ*x* and *D* represents the distance from the current site to the center.

**Figure 2:**
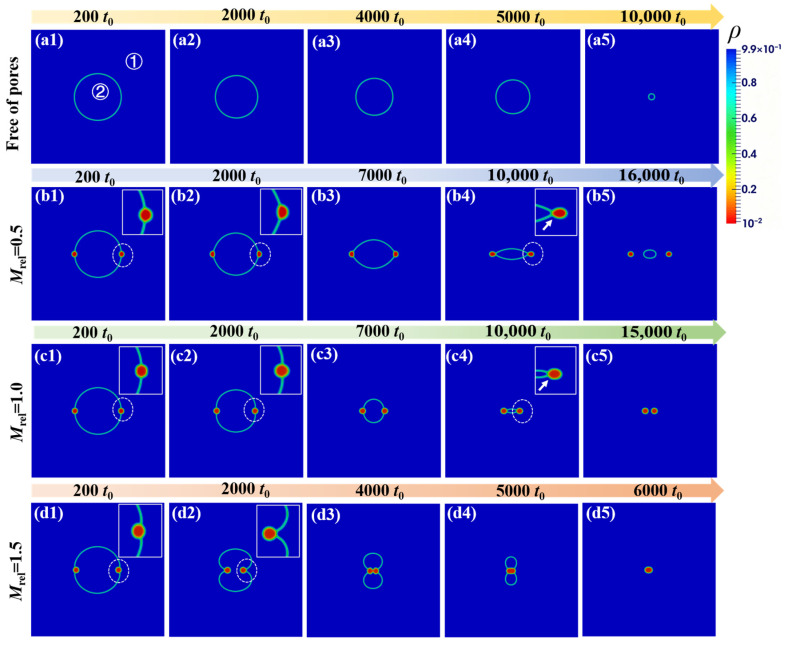
Snapshots of the collective migration of pores and GBs with different relative mobilities of pores and GBs. (**a1**–**a5**) free of pores on GBs; (**b1**–**b5**) *M*_rel_ = 0.5; (**c1**–**c5**) *M*_rel_ = 1.0 and (**d1**–**d5**) *M*_rel_ = 1.5. In (**a1**), ① and ② denote the two grains. Zoomed insets highlight the early-stage pore-shape change in panels (**b1**,**c1**,**d1**), and the pore-detachment events in panels (**b4**,**c4**), with the moment of detachment marked by arrows.

Since pore-induced pinning or drag is governed by the mobility contrast between pores and GBs [14], the relative mobility is expected to play a central role in determining GB migration rates and grain-growth behavior. Therefore, in our work, we introduce a relative mobility ratio *M*_rel_ = *M_p_/M_b_*, where *M_p_* and *M_b_* are the pores and GB mobilities, respectively. A small *M*_rel_ value indicates low pore mobility, whereas a large value indicates high pore mobility. The grain growth kinetic behaviors involving pore migration are investigated under various relative mobility conditions by assigning different *M*_rel_ values. Snapshots of the morphologies for *M*_rel_ = 0.5, 1.0, and 1.5 are depicted in Figure 2. The bi-crystal system comprises a circular grain with a radius of 25Δ*x* embedded in a larger grain; the outer one is designated as grain 1 and the inner one as grain 2. Two oppositely positioned pores with a radius of 5Δ*x* are preset on the GB. The curvature is equivalent everywhere on the boundary of grain 1 and grain 2, the two pores attached on the equivalent positions suffering equal force from temperature gradient and GB curvature. It should be noted that although the initial relative mobility (*M*_rel_) has been pre-assigned, the rate of pores and GBs is varying according to local temperature, temperature gradient, and curvature.

Grain 1 grows at the expense of grain 2 as a result of the inward migration of GBs at various speeds, as illustrated in Figure 2. In Figure 2(a1–a5), the pore-free GB moves inward into grain 2, driven by surface energy reduction, leading to a steady shrinkage rate of grain 2 while maintaining its circular shape. In Figure 2(b1–b4), with a *M*_rel_ = 0.5 (low-mobility pores), the two pores exert a pinning force on GB migration, causing the GBs segments pinned by the pores to migrate at a slower rate than the unpinned segments. As a result, the initially circular grain 2 evolving into a lenticular shape (Figure 2(b4)). Over time, the highly mobile GBs break away from the pinning effect of pores, leaving the pores behind, and grain coalescence occurs prior to pore coalescence, as shown in Figure 2(b5). The detachment of pores from GBs facilitates the restoration of grain 2 from a lenticular shape to a circular shape. In contrast to the cases of pore-free (Figure 2(a1–a5)), the delayed annihilation of grain 2 is attributed to the pinning effect of pores on GBs.

For *M*_rel_ = 1, where the mobility of pores and GBs are comparable, both pores and GBs migrate concurrently, as shown in Figure 2(c1–c5). In this situation, grain 2 shrinks while maintaining its circular shape. A comparison of the morphological snapshots at 4000 *t*_0_ and 5000 *t*_0_ reveals that grain 2 shrinks at a rate comparable to that in the pore-free case, which is markedly faster than in the *M*_rel_ = 0.5 case, leading to its much earlier annihilation. This indicates that the presence of mobile pores—with mobility comparable to that of GBs—accelerates the coarsening of grain 1 by exerting a dragging force on GB migration, leading to a quicker shrinkage and earlier annihilation of grain 2 compared with the low-mobility cases.

For *M*_rel_ = 1.5, where pores are highly mobile, the evolution of the pore–GB complex is depicted in Figure 2(d1–d5). Under this condition, the GB segments attached to pores move inward much faster than other segments. This leads to the transformation of the initial circular shape of the inner grain into two distinct semi-circles and eventually causes its splitting into two grains. Furthermore, the coalescence of pores occurs prior to grain coalescence. Although the pores are highly mobile, they persistently drag the GBs along without detaching. The fast-moving pores exert a continuous dragging force on the GBs as long as they remain attached. This accelerates directional coarsening of grain 1 along the pore migration direction and promotes the annihilation of grain 2.

Overall, the mobile pores exert a progressive impact on grain growth. Low-mobility pores have a pinning effect on the migration of GBs, thereby inhibiting grain growth. Conversely, highly mobile pores can drag GBs to migrate together, thus accelerating grain growth. To some extent, the grain microstructures, which are controlled by GB migration behavior, depend on the migration of pores. For instance, in UO_2_ fuel rods, the columnar grains surrounding the central cavity are associated with directional pore migration [24,27].

**Figure 3:**
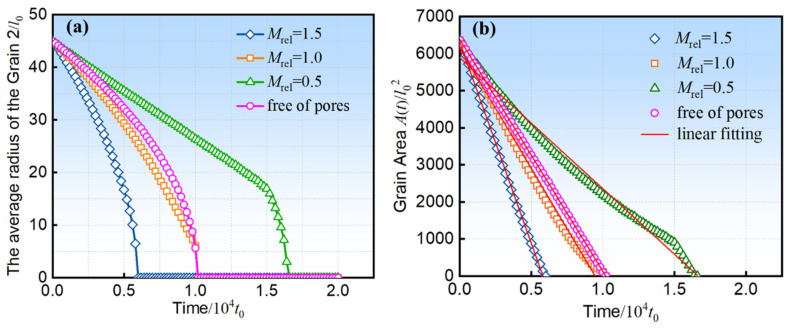
The shrinkage rate of grain 2 under relative mobilities *M*_rel_ are 0.5, 1.0, 1.5, respectively, and pore-free. (**a**) Mean grain radius over time. (**b**) Grain area over time.

Figure 3 presents a quantitative analysis of the impact of pores on the radius and grain area of grain 2, based on the data from Figure 2. The pore-free case is used as a reference. As shown in Figure 3a, the mean grain radius of grain 2 shrinks at various rates depending on the relative mobility *M*_rel_. For *M*_rel_ = 0.5, the grain radius shrinks more slowly than in the pore-free case; for *M*_rel_ = 1, the shrinkage rate is comparable to that of the pore-free case; for *M*_rel_ = 1.5, grain 2 shrinks drastically. Pores with high mobility facilitate GB migration and grain coarsening, whereas those with low mobility hinder coarsening by restricting GB migration.

For *M*_rel_ = 0.5 in Figure 3a, the grain radius curves are piecewise linear, depending on whether the pores are attached to or detached from the GBs. When GBs are attached with pores, grain 2 shrinks slowly due to the pinning effect of the pores; when pores detach from GBs, grain 2 shrinks rapidly as a result of GB area reduction. Over time, the mechanism of grain growth shifts from pore-controlled to boundary-controlled, as confirmed by studies on the sintering of porous ceramics [32].

In general, the average grain area *A*(*t*) follows a parabolic growth law for ideal grain growth in dense polycrystals [33]:

(15) A(t)−A(0)=kt

(16) $$k=2\pi \gamma_{gb}M_{b}=2\pi L\kappa_{\alpha \beta }$$

where *A*(0) represents the initial grain area. *k* is the rate constant related to the migration characteristics of GBs as given by Equation (16). It can be observed that *A*(*t*) varies linearly with time *t*, which is the manifestation of the parabolic growth law.

Relationships between the statistical average grain 2’s area and time *t* are shown in Figure 3b, under various *M*_rel_ values. It is seen that the area reduces linearly over time, except for the *M*_rel_ = 0.5 case, which exhibits two piecewise linear segments due to the shift in grain growth mechanism from pore-control to boundary-control.

### 3.3. Impact of Mobile Triple-Junction Pores on Grain Growth

**Figure 4:**
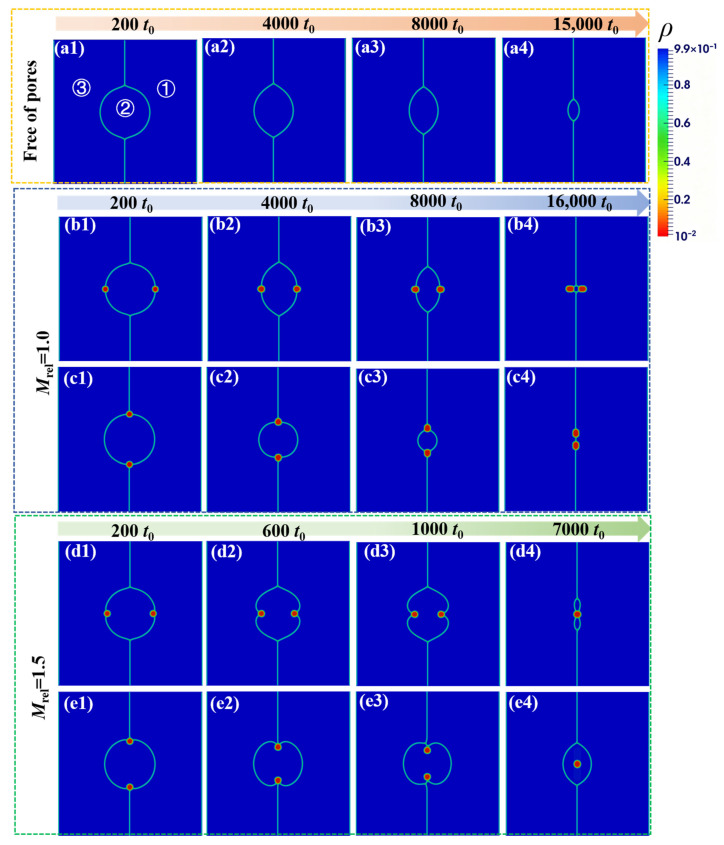
Snapshots of the collective migration of pores and GBs with the pores located at adjoining boundaries and triple junctions under two different relative mobilities: (**a1**–**a4**) grain growth free of pores. (**b1**–**b4**,**c1**–**c4**) for a relative mobility of *M*_rel_ = 1.0, (**d1**–**d4**,**e1**–**e4**) for a relative mobility of *M*_rel_ = 1.5.

**Figure 5:**
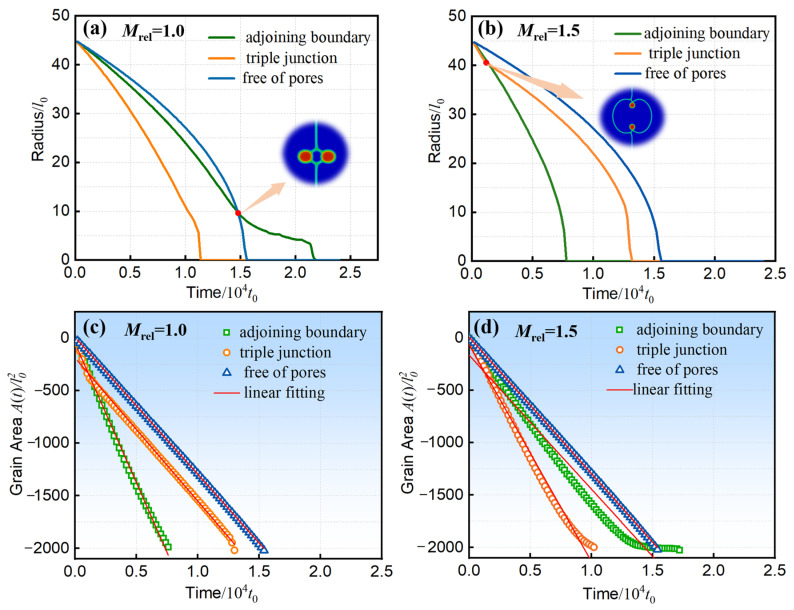
Evolution of grain 2’s radius and area with attached pores at adjoining boundaries and triple junctions under relative mobilities *M*_rel_ (pores to GBs) of 1.0 and 1.5. (**a**,**b**) represent the mean grain radius, while (**c**,**d**) show the mean grain area.

Mobile triple-junction pores significantly influence grain growth, as triple junctions are preferred sites for vacancy accumulation and pore formation. To explore this, Figure 4 compares pores at triple junctions with those at adjoining boundaries. Triple junctions, where three GBs meet, exhibit lower mobility than adjoining boundaries, leading to triple-junction pinning. Figure 4 illustrates a three-grain system with a circular grain 2 between grains 1 and 3 under a central temperature field and uniform GB energy. The analysis of *M*_rel_ = 1.0 and 1.5 is shown in Figure 5.

Figure 4(a1–a4) present the pore-free samples as a reference. In the absence of pores, grain 2 shrinks from circular to ellipsoidal, with its long axis aligning with the two triple junctions. This indicates finite triple-junction mobility in contrast to the adjoining boundaries, consistent with the experimental and numerical results [34,35].

For *M*_rel_ = 1, Figure 4(b1–b4,c1–c4) show adjoining-boundary and triple-junction pores, respectively. For adjoining-boundary pores, grain 2 shrinks similarly to the pore-free case, with annihilation coinciding with pore coalescence. In contrast, for triple-junction pores, grain 2 shrinks and is annihilated before pore coalescence. GB migration and grain evolution are significantly influenced by mobile pores, but the effects on triple junctions and adjoining boundaries differ. Figure 5a shows grain 2’s mean radius, with complete annihilation at 23,000 *t*_0_ (adjoining-boundary), 12,000 *t*_0_ (triple junction), and 15,500 *t*_0_ (pore-free).

For *M*_rel_ = 1.5, Figure 4(d1–d4,e1–e4) show adjoining-boundary and triple-junction pores, respectively. Highly mobile pores drag GBs to migrate together, resulting in the bending and extension of GBs, as indicated by the curved pore migration traces. Pores at adjoining-boundary GBs are harder to detach, while those at triple junctions break away from GBs, causing pore coalescence to occur before grain 2 is fully annihilated (Figure 4(d4,e4)). These observations agree with the pore–GB detachment mechanism described by Petrishcheva et al. [30]. Figure 5b shows grain 2’s mean radius, with complete annihilation at 8000 *t*_0_ (adjoining-boundary), 13,000 *t*_0_ (triple junction), and 15,500 *t*_0_ (pore-free).

Figure 5c,d present the evolution of the mean area of grain 2 for *M*_rel_ = 1.0 and 1.5, respectively. In the absence of pores, grain 2 shrinks linearly over time, which is in accordance with the parabolic growth law as the linear relation between *A*(*t*) and *t* as anticipated by Equation (13) developed for dense polycrystalline. In the presence of pores (adjoining-boundary or triple junctions), the shrinkage rate is apparently accelerated, indicating that highly mobile pores promote grain evolution. When pores are located at adjoining boundaries, the contraction rate of grain 2 is significantly higher for *M*_rel_ = 1.5 than for *M*_rel_ = 1.0. In contrast, when pores are located at triple-junctions, the shrinkage rate of grain 2 for *M*_rel_ = 1.5 is lower than that for *M*_rel_ = 1.0. Pore configuration and mobility are crucial for grain growth dynamics, and grain microstructures depend on the complex interaction of pores with either adjoining boundaries or triple junctions.

## 4. Discussion

### 4.1. Pinning Effect of Immobile Pores on GBs

The results presented above illustrate the collective migration behavior between the mobile pores and GBs. The GB migration and the grain microstructures can thus be accounted for by this collective migration behavior. A migration model for GBs decorated with pores has been proposed based on the force balance between the surface tension of GBs and the pinning effect of immobile pores, as described by the Zener model [13,14]. The GB migration rate is given by:

(17) $$v_{b}=M_{b}(F_{b}-N_{p}F_{p})$$

where $M_{b}$ is the intrinsic mobility of GBs, $N_{p}$ is the number of pores per unit area (i.e., areal density of pores), and $F_{p}$ is the resistance exerted by a pore on the GBs. The surface tension of GBs is given by $F_{b}=\alpha \gamma_{gb}/R_{g}$ in the absence of obstacles [36], where α is a geometric factor, $\gamma_{gb}$ is the interfacial energy of grain boundary, and *R_g_* is grain radius. Accordingly, two possible scenarios may occur: either the GBs break free and leave the pores behind when $F_{b}\gg N_{p}F_{p}$ or the GBs are pinned by pores when $F_{b}=N_{p}F_{p}$ [36].

**Figure 6:**
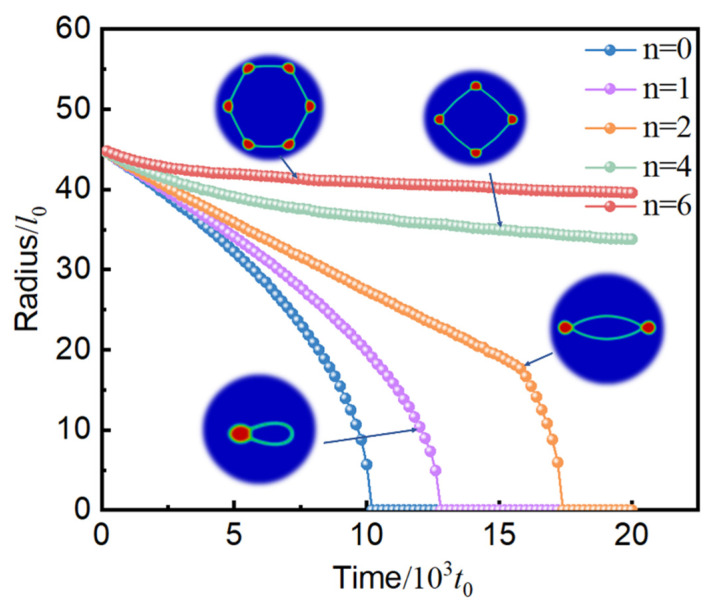
Evolution of grain 2’s mean grain radius with different immobile pore densities (*n* denotes the number of pores on grain boundaries).

The phase field simulation results of grain evolution under different areal densities of immobile pores are shown in Figure 6. Compared with the pore-free case (*n* = 0), immobile pores impede GB migration. As the density of immobile pores increases, the shrinkage of grain 2 becomes slower, indicating that the migration rate of GBs is reduced. The pinning effect of immobile pores on GB migration is consistent with the Zener model as described by Equation (15).

### 4.2. Concurrent Migration of Pore–GB Complex at an Equal Rate

Nichols extended the immobile pore model by adopting the simplifying assumption that pores remain attached to GBs and migrate at the same rate (i.e., $v_{p}$= $v_{b}$). This is expressed by reformulating Equation (17) as:

(18) $$M_{p}F_{p}=M_{b}(F_{b}-N_{p}F_{p})$$

where $v_{p}$ = $M_{p}F_{p}$ is the average pore rate, and $M_{p}$ is the pore mobility. Rearranging Equation (18) yields

(19) $$v=\frac{M_{p}M_{b}}{M_{p}+N_{p}M_{b}}F_{b}$$

From Equation (19), two limiting cases can be derived.

The first limit is that when *M_p_* ≫ *N_p_M_b_*, the rate of the pore–GB complex becomes *M_b_F_b_* (i.e., *v* ≈ *M_b_F_b_*). The condition of *M_p_* ≫ *N_p_M_b_* demands a large pore mobility (*M_p_*) and a small GB mobility (*M_b_*), indicating a high relative mobility (*M*_rel_). In this case, the highly mobile pores can keep pace with the GB, so the GB migration rate is barely affected by pores. Thus, the GB migration rate is mainly controlled by GB mobility, which is known as the boundary-controlled mechanism.

The second limit is that when *M_p_* ≪ *N_p_M_b_*, the rate of the pore–GB complex is approximately (*M_p_*/*N_p_*)*F_b_*. The condition of *M_p_* ≪ *N_p_M_b_* requires a small pore mobility (*M_p_*) and a large GB mobility (*M_b_*), indicating a low relative mobility (*M*_rel_). In this case, the GB migration rate is proportional to the pore mobility (*M_p_*) and inversely proportional to the pore density (*N_p_*). Here, the GB migration rate is mainly controlled by pores, a situation known as the pore-controlled mechanism.

The migration rate of the pore–GB complex is dependent on GB mobility, pore mobility, pore density, and GB curvature, as discussed above. The phase field simulation results as presented in Figure 2 and Figure 4 demonstrate the collective migration behavior under various conditions for a given pore density. In Figure 2 and Figure 4, the pore–GB complexes migrate concurrently at equal rates. In some cases (e.g., Figure 2(c1–c5) with *M*_rel_ = 1.0, Figure 4(b1–b4) with *M*_rel_ = 0.5), pores and GBs migrate together until grain coalescence is complete. In some cases (e.g., Figure 2(b1–b5) with *M*_rel_ = 0.5), pores and GBs may initially migrate together, but GBs escape from the pinning of the pores because GBs migrate faster than pores. These scenarios approximate the second limit, where pore-controlled migration predominates before pore–GB separation.

According to the above two limits, all conditions fall between two extremes: low pore mobility and high pore mobility. For the extreme of low pore mobility, the strong pinning effect slows down GB migration, approaching the second limit (pore-controlled mechanism). For the extreme of high pore mobility, the pinning effect weakens, and GB migration is minimally affected by pores, transitioning to the first limit (boundary-controlled mechanism). However, phase field simulations show deviations from the first limit when pores are highly mobile. For example, with *M*_rel_ = 1.5 (highly mobile pore), the grain size changes in Figure 3 and Figure 5 indicate that these highly mobile intergranular pores significantly accelerate GB migration, exceeding the rate of bare GBs. In this situation, the pore-controlled mechanism of GB migration still operates, contrary to the traditional view that the boundary-controlled mechanism dominates with enhanced pore mobility. Moreover, pore–GB separation occurs, but it is the pores that break away from the binding of the GB, leaving the GB behind, as shown in Figure 4(e1–e4) (*M*_rel_ = 1.5); subsequently, pore coalescence takes place before grain coalescence.

This accelerated GB migration due to highly mobile pores, which promotes grain coarsening, is not accounted for by Equation (17). Nevertheless, such a situation is quite likely to occur in UO_2_ fuels under severe environmental conditions, and therefore, the microstructures of UO_2_ fuels are significantly affected by the mobility and direction of these highly mobile pores—a phenomenon not previously reported. This does not violate the Zener and Nichols framework. The pore generates no driving force of its own; it acts only as a conduit that transfers the external thermal-gradient force to the GB. The acceleration is therefore externally driven, not intrinsic. Our results reduce to the classical picture in the appropriate limit, as is evident directly from the model equations.

### 4.3. Accelerated Migration of GBs Due to Highly Mobile Pores

To clarify the impact of highly mobile pores on GB migration, a more sophisticated approach is employed to describe the migration of GBs in a pore–GB complex system, taking into account the rate disparities between GBs and pores. This can be expressed as follows

(20) $$v_{b}-v_{p}=M_{b}(F_{b}-N_{p}F_{p})$$

Substituting $v_{p}=M_{p}F_{p}$ into Equation (19), the GB migration rate becomes:

(21) $$v_{b}=M_{b}F_{b}-(N_{p}M_{b}F_{p}-M_{p}F_{p})=M_{b}F_{b}+(M_{p}-N_{p}M_{b})F_{p}$$

The second item on the right-hand side represents the contribution of mobile pores to GB migration. If the value in the parentheses of the second term is negative, pores slow the GB migration rate (pinning effect), while a positive value accelerates it (dragging effect). This depends on pore density (*N_p_*) and mobility (*M_p_*) for a given GB mobility (*M_b_*). Therefore, it can be deduced that higher pore density and lower relative mobility (*M*_rel_) tend to yield a negative value, indicating a pinning effect. Conversely, lower pore density and higher *M*_rel_ tend to yield a positive value, indicating a dragging effect. Thus, for a given pore density (*N_p_*), pore effects on GB migration fall into two categories: pinning or dragging.

In cases where *M_p_/(N_p_M_b_)* = *M*_rel_*/N_p_* > 1, corresponding to a high relative mobility *M*_rel_ of the pores, the highly mobile pores drag the GBs along, facilitating their migration. This is supported by the phase field results for *M*_rel_ = 1.5, as shown in Figure 3 and Figure 5.

On the other hand, when *M*_rel_*/N_p_* < 1, indicating low pore mobility, the slower-moving pores slow down the GB migration. For *M*_rel_ = 0.5, as seen in Figure 3 and Figure 5, slow mobile pores pin GBs, delaying grain annihilation.

This rate-disparity model, together with the pore density *N_p_*, provides a mechanistic basis for the pinning/dragging competition. Section 4.4 introduces a complementary, rate-based ratio *R_v_* = *M*_rel_·*F*_rel_, which captures the same competition from the perspective of relative migration rates rather than pore density, without requiring an explicit measure of *N_p_*. This ratio reorganizes the pinning/dragging dichotomy here into three regimes: pinning, weak interaction, and pore-assisted migration.

**Figure 7:**
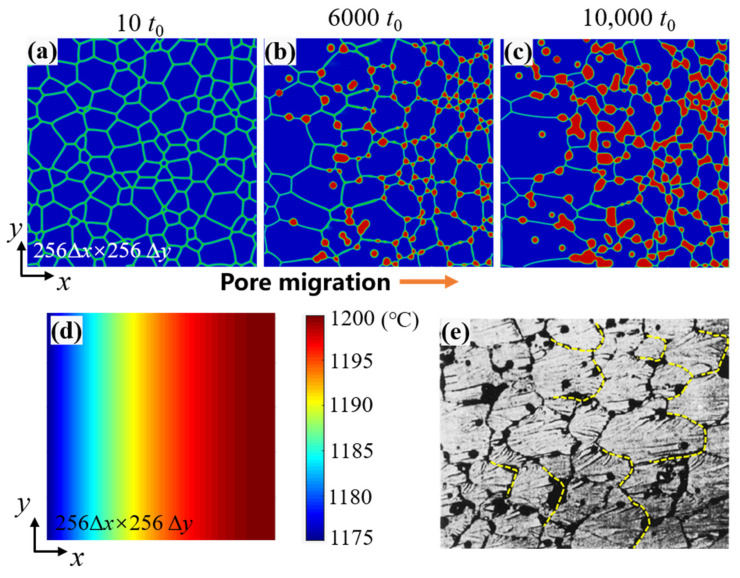
(**a**–**c**) The synergistic evolution of pore–grain boundary interactions in polycrystalline structures under temperature gradient. (**d**) Temperature field distribution. (**e**) Migration of fission-gas bubbles up the thermal gradient in a (Pu_0.2_, U_0.8_)O_2_ fuel rod. Temperature increases to the right [37].

To clarify the regulating effect of highly mobile pores on GB migration under a temperature gradient, we simulate the synergistic evolution of pore–GB interactions in polycrystalline structures (Figure 7a–c). A parabolic temperature field was applied along the *x*-direction, Figure 7d, coupled with continuous irradiation: pores nucleated spontaneously at grain boundaries; and migrated toward the high-temperature region on the right side under the temperature gradient, as indicated by the orange arrows. In the high-temperature zone, where the temperature gradient is lower, pore migration is slow, and grains retained an equiaxed morphology. In the low-temperature zone, where the temperature gradient was steeper, highly mobile pores promoted directional grain growth along the temperature gradient, and the grain boundaries bent synchronously in the same direction. This behavior agrees with the evolution shown in Figure 7e: pores migrate rightward along the temperature gradient and cause the GBs to bend [37]. This further demonstrates that pore–GB coupling under a temperature gradient can strongly affect grain-shape evolution. The temperature gradient provides the driving force for vacancy and matrix–atom diffusion within pores, as well as atomic transport across the GBs, which in turn induces grain-boundary bowing. Mechanistically, pore migration can drag grain boundaries to produce directional boundary motion under a temperature gradient. This coupled response is a deformation process governed by diffusion and interfacial migration, and it is closely related to thermal creep [38].

### 4.4. M_rel_-F_rel_ Regime Map: From Pinning to Pore-Assisted Migration

The above discussion reveals that the migration rates of GBs and the associated microstructures are closely related to the mobile pores attached to GBs. Phase-field simulations are capable of capturing the subtle evolution of pore morphology resulting from pore–GB interactions, as well as the spatiotemporal migration behavior of pore–GB complexes under various conditions. This approach outperforms conventional analytical models, such as the Zener model (Equation (15)), which assumes immobile pores, or Equation (17), which assumes equal migration rates for pores and GBs. It also exceeds models that consider the rate differences between GBs and pores (Equation (19)) but assume pores to be rigid circular bodies.

The relative mobility (*M*_rel_) of pores and GBs determines, to some extent, whether they migrate concurrently as pore–GB complexes or separately. During concurrent migration, the highly mobile component drags the slower one, while the slower component pins the faster one. Whether the effect is pinning or dragging depends on the local morphology and varies over time and position, making quantitative experimental or analytical characterization challenging.

In Section 4.1, Section 4.2 and Section 4.3, we have discussed the analytical models of GB migration: (1) pinning by immobile pores (the classical Zener limit); (2) concurrent migration of pore–GB complexes under pinning by mobile pores; (3) concurrent migration of pore–GB complexes with pore-assisted acceleration by highly mobile pores. A comparison of these analytical models with phase field simulation results is also included. While analytical models focus on pinning by immobile or low-mobility pores—consistent with simulations—they rarely address cases where pore migration exceeds GB migration. This scenario is crucial in extreme circumstances, such as in UO_2_ nuclear fuels, where columnar grains formed near the fuel pellet centerline under operating conditions are associated with highly mobile pores. Our previous studies also confirm that columnar grains in UO_2_ fuels form only when pore migration exceeds GB migration [31]. However, the role of highly mobile pores in GB migration and microstructure evolution remains poorly understood.

The simulation results presented in this work highlight the significant role of the pore-to-GB mobility ratio *M*_rel_ in the transition from pore-induced pinning to pore-assisted migration of GBs. High pore mobility can accelerate GB migration, but the extent of this acceleration also depends on the local GB driving force: when the local GB migration rate is very low, the accelerating effect of the pore is limited. This indicates that the pore effect is not determined by mobility alone but by the competition between the pore and GB migration rates, characterized by their ratio *R_v_* = *v_p_*/*v_b_* = *M*_rel_·*F*_rel_, where *F*_rel_ *= F_p_/F_b_* is the relative driving force. The average GB driving force is $F_{b}=\alpha \gamma_{gb}/R_{g}$, where *α* is a geometric factor (*α* = 2 for a spherical grain), $\gamma_{gb}$ is the GB interfacial energy [35], and *R_g_* the grain radius; *F_b_* therefore evolves as the grain shrinks. The pore driving force *F_p_* is given by Equation (9). Since *F_p_* is fixed by the imposed temperature gradient while *F_b_* varies with curvature, *F*_rel_ is not an independently prescribed quantity; in the present simulations the controlled variable is *M*_rel_, which, at a fixed temperature gradient, monotonically represents *R_v_*.

(22) $$F_{p}=\Delta H_{vap}p_{0}\exp\left(-\frac{\Delta H_{vap}}{RT}\right){\left(\frac{dT}{dr}\right)}_{p}$$

**Figure 8:**
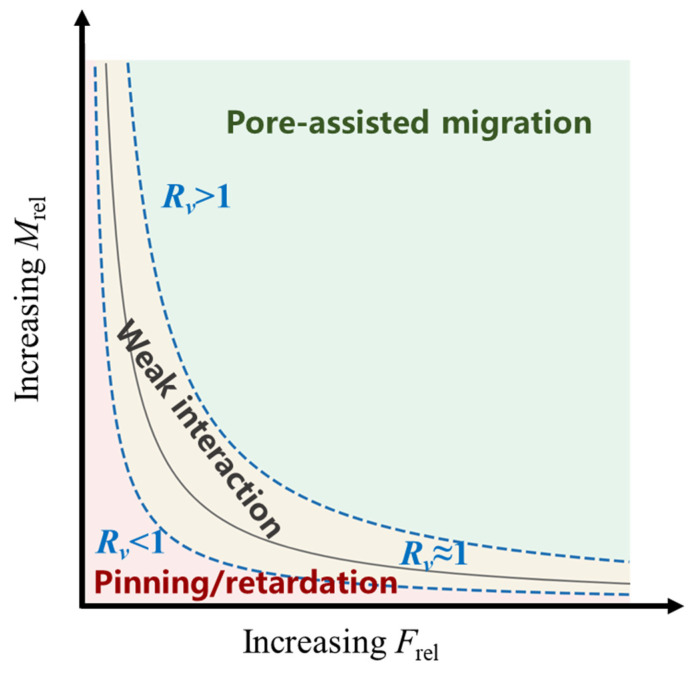
Schematic regime map in the *M*_rel_-*F*_rel_ plane. Regime boundaries correspond to the analytically defined ratio *R_v_* = *v_p_*/*v_b_* = *M*_rel_·*F*_rel_ (Equation (22) and the associated GB driving-force expression), with *R_v_* > 1, *R_v_* ≈ 1,and *R_v_* < 1 corresponding to pore-assisted migration, weak interaction, and pinning/retardation, respectively. Because *F*_rel_ evolves self-consistently with grain curvature rather than being an independently prescribed simulation variable, the boundary curves are drawn schematically; simulations varied *M*_rel_ at fixed temperature gradient, which monotonically represents *R_v_*.

On this basis, the *M*_rel_-*F*_rel_ map (as shown in Figure 8) is organized into three regimes according to *R_v_*:

- Pore-assisted migration (*R_v_* > 1): sufficiently mobile pores remain attached and, driven by the temperature gradient, entrain the GB, accelerating its migration beyond the bare GB rate.
- Weak interaction (*R_v_* ≈ 1): In this moderate *M*_rel_ region, pores have minimal effect on GBs, allowing for concurrent migration without significant interaction.
- Pinning/retardation (*R_v_* < 1): slower-moving pores exert a resistive (drag) effect that retards GB migration below the bare rate. When *R_v_* departs far from unity, pore–GB separation may occur.

In the schematic map of Figure 8, the regime is set by *R_v_* = *M*_rel_·*F*_rel_, such that neither the mobility ratio nor the driving-force ratio alone determines the response—both act together. Because *F_p_* is fixed by the imposed temperature gradient, variations in *F*_rel_ *= F_p_/F_b_* mainly reflect changes in the GB driving force *F_b_*, which increases as the grain shrinks ($F_{b}=\alpha \gamma_{gb}/R_{g}$). Thus, at a given *M*_rel_, a larger *F*_rel_ (smaller *F_b_*) raises *R_v_* and shifts the system toward a more pore-dominated response, increasing the extent of pore-assisted acceleration. Conversely, a smaller *F*_rel_ decreases *R_v_* toward pore-induced retardation. In the present simulations, where the temperature gradient is fixed and *M*_rel_ is the controlled variable, *M*_rel_ monotonically represents *R_v_*. Thus, the transition from pinning through weak interaction to pore-assisted migration is captured by increasing *M*_rel_.

## 5. Conclusions

In this study, we employ the phase field method to examine the effect of intergranular mobile pores on GB migration under a temperature gradient. The following main conclusions can be drawn:

1. Beyond the classical pinning effect, the simulations reveal a pore-assisted acceleration regime under a temperature gradient. The pore effect is governed not by the mobility ratio alone but by the pore-to-GB migration-rate ratio *R_v_* = *M*_rel_·*F*_rel_, which combines the relative mobility and the relative driving force. When *R_v_* > 1, the sufficiently mobile pore entrains the boundary, induces GB bending, and raises the net GB velocity above the bare GB rate (pore-assisted migration); for *R_v_* < 1, the lagging pore exerts a drag that pins the boundary and reduces its migration; *R_v_* ≈ 1 corresponds to weak interaction. The resulting *M*_rel_-*F*_rel_ map provides a mechanism-based framework for distinguishing these regimes and for interpreting the competition between pore-driven and boundary-driven migration.
2. When pores remain attached and co-migrate with the GB, the kinetics are rate-limited by the slower of pore transport and boundary migration. For *R_v_* ≥ 1, the pore keeps pace with the boundary, the evolution remains GB-controlled, and the grain area follows the near-linear von Neumann–Mullins kinetics. For *R_v_* < 1, pore transport becomes the bottleneck. The attached pore imposes a drag, the evolution becomes pore-controlled, and the GB velocity drops markedly, deviating clearly from this linear behavior.

## Figures and Tables

**Table 1:** Parameters used in the phase field simulations [27].

Symbol	Names	Values
$a_{i}$	Coefficients in the transport rate of Equation (9)	*a*_1_ = 5 × 10^−16^, *a*_2_ = 0.988, *a*_3_ = 6.395 × 10^−6^, *a*_4_ = 3.513 × 10^−9^, *a*_5_ = 3.0 × 10^−12^ [28]
*R*	The ideal gas constant	8.314 J/(mol K)
$\Delta H_{\nu ap}$	Vaporization enthalpy	5.8 × 10^5^ J/mol [28]
*p*_0_	The pressure prefactor	4.359 × 10^11^ Pa [28]
$\gamma_{gb}$	Grain boundary energy	0.289 J/m^2^ [28]
*W*	Interface width	4∆*x*
*M* *_b_*	Grain boundary mobility	8.01 m^4^/J s [29]

## Data Availability

The original contributions presented in this study are included in the article. Further inquiries can be directed to the corresponding authors.
